# Evolutionary divergence in life history traits among populations of the Lake Malawi cichlid fish *Astatotilapia calliptera*

**DOI:** 10.1002/ece3.3311

**Published:** 2017-09-12

**Authors:** Paul J. Parsons, Jon R. Bridle, Lukas Rüber, Martin J. Genner

**Affiliations:** ^1^ School of Biological Sciences University of Bristol Bristol UK; ^2^ University of Exeter Penryn UK; ^3^ Naturhistorisches Museum der Burgergemeinde Bern Bern Switzerland; ^4^ Institute of Ecology and Evolution University of Bern Bern Switzerland

**Keywords:** adaptive radiation, egg size, *F*_ST_‐*Q*_ST_, growth rate, phenotypic plasticity

## Abstract

During the early stages of adaptive radiation, populations diverge in life history traits such as egg size and growth rates, in addition to eco‐morphological and behavioral characteristics. However, there are few studies of life history divergence within ongoing adaptive radiations. Here, we studied *Astatotilapia calliptera*, a maternal mouthbrooding cichlid fish within the Lake Malawi haplochromine radiation. This species occupies a rich diversity of habitats, including the main body of Lake Malawi, as well as peripheral rivers and shallow lakes. We used common garden experiments to test for life history divergence among populations, focussing on clutch size, duration of incubation, egg mass, offspring size, and growth rates. In a first experiment, we found significant differences among populations in average clutch size and egg mass, and larger clutches were associated with smaller eggs. In a second experiment, we found significant differences among populations in brood size, duration of incubation, juvenile length when released, and growth rates. Larger broods were associated with smaller juveniles when released and shorter incubation times. Although juvenile growth rates differed between populations, these were not strongly related to initial size on release. Overall, differences in life history characters among populations were not predicted by major habitat classifications (Lake Malawi or peripheral habitats) or population genetic divergence (microsatellite‐based *F*_ST_). We suggest that the observed patterns are consistent with local selective forces driving the observed patterns of trait divergence. The results provide strong evidence of evolutionary divergence and covariance of life history traits among populations within a radiating cichlid species, highlighting opportunities for further work to identify the processes driving the observed divergence.

## 1 INTRODUCTION

Adaptive radiation is characterized by the rapid evolution of ecologically differentiated species that share recent common ancestry (Schluter, 2000). Although life history traits can diverge among derived species within radiations (Duponchelle, Paradis, Ribbink, & Turner, 2008), the role of local life history adaptation in restricting gene flow among populations remains far less well‐understood than adaptation in eco‐morphological and behavioral traits. This is surprising, given that many studies have demonstrated that intraspecific variation in life history strategies is driven by local environmental variation, including the quality of the food (Segers & Taborsky, 2011), habitat availability (Rollinson & Hutchings, 2013), and key limits to reproduction including local predation regimes (Segers & Taborsky, 2012). It is, therefore, important to assess the role of life history traits, and potential constraints in their evolution, during the process of adaptive radiation.

Studies of life history evolution often focus on how spatial or temporal variation in the environment can drive selection to optimize fitness in traits such as offspring size, offspring number, and growth rates. It is generally assumed that larger offspring must be fitter, and empirical data often support this view (Bashey, 2008; Hutchings, 1991; Reznick, Bryga, & Endler, 1990; Riesch, Plath, & Schlupp, 2012; Sogard, 1997). Therefore, we should expect individual females to favor larger offspring wherever possible. However, the energy required for somatic maintenance means that only a portion of the resources of any female can be allocated for reproduction. Selection should, therefore, modify the balance of offspring size to offspring number depending on the resources available, and show spatial variation among habitats. How selection operates on such traits, however, will be determined not only by readily measurable spatial contrasts in habitat characteristics, but also by habitat predictability (Morrongiello, Bond, Crook, & Wong, 2012), and the extent of plasticity in the trait (e.g., Burgess & Marshall, 2014).

Life history traits show strong covariance, so evidence that habitat predictability and resource availability can both drive selection on life history traits (e.g., Winemiller & Rose, 1992). Winemiller (2005) suggests that these factors may slow down, or prevent, local adaptation in some habitats, but will accelerate local adaptation (and adaptive radiation) in others. Given this background, in this study we used the mouthbrooding cichlid fish *Astatotilapia calliptera* to take the first steps to investigate population level divergence, and covariance in life history traits, within the context of cichlid adaptive radiation. The species is useful for studying life history evolution, on account of the considerable maternal care exhibited (Konings, 2007; Ribbink, 1990), with females collecting eggs after fertilization and incubating them in their mouths (Ribbink, 1990).

*Astatotilapia calliptera* is part of the Lake Malawi haplochromine radiation (Malinsky et al., 2017) but, unlike the other members of the flock that are lacustrine specialists it is a generalist, occupying both the littoral margins of Lake Malawi and peripheral habitats including rivers and shallow lakes. The main body of Lake Malawi is comparatively stable, with relatively minor changes in water level between seasons and over decadal timescales (Scholz et al., 2011). By contrast, peripheral water bodies are prone to both flooding in the wet season and drought or even complete habitat desiccation (e.g., Nicholson, 1998; for Lake Chilwa) in the dry season (Kingdon, Bootsma, Mwita, Mwichande, & Hecky, 1999; Pauw, Thurlow, & Van Seventer, 2010). This strong seasonal variability in water availability leads to associated changes in habitat productivity, thermal regime, and oxygen availability. The species also represents a useful model when considering evolutionary processes during early‐stage adaptive diversification. The species has seeded a sympatric species pair within a crater lake (Malinsky et al., 2015) and has also been proposed to have taken a role in generating the main species radiation in Lake Malawi (Malinsky et al., 2017). Importantly the species exhibits population variation in male color and eco‐morphological traits. These differences are associated with assortative mating suggestive of incipient speciation in both allopatry (Nichols et al., 2015; Tyers & Turner, 2013), and sympatry (along a depth cline, Malinsky et al., 2015).

In this study, we used common garden laboratory‐based experiments to test whether populations of *A. calliptera* differ in clutch size, egg mass, brooding duration, and the speed of early growth. We also tested if the observed variation differs predictably between Lake Malawi and peripheral water bodies, and how evolutionary divergence is associated with trade‐offs among life history traits. We expected that occupants of lacustrine sites should possess traits that promote intraspecific competitiveness, namely small broods of larger offspring. In contrast we expected that populations from riverine sites should possess traits that maximize productivity, namely larger broods of smaller offspring. We also assessed the role of selection relative to genetic drift by exploring the relationship between population‐level phenotypic divergence of life history traits (*Q*_ST_) and population‐level genetic divergence (*F*_ST_), estimated using allelic variation at microsatellite loci. We predicted that if strong local adaptation in life history traits was taking place, *Q*_ST_ would be independent of genetic distance between sampling sites.

## 2 MATERIALS AND METHODS

### 2.1 Collection and animal husbandry

**Figure 1:**
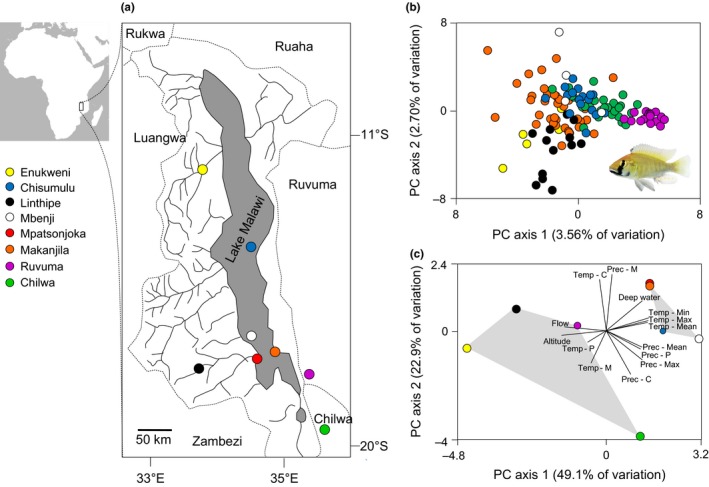
(a) Locations of *Astatotilapia calliptera* populations in Malawi studied. For coordinates see Table 1. (b) Ordination of genetic structure among individuals from seven of the studied populations, based on seven microsatellite loci (Table 2). (c) Principal Component Analysis of environmental similarity of source locations, showing Lake Malawi populations (upper right) and peripheral habitat populations (lower left). Labeled lines indicate associations between the labeled variable and the axis of variation. Temp–Max (maximum monthly temperature), Temp–Min (minimum monthly temperature), Temp–Mean (average monthly temperature), Temp‐C (temperature constancy), Temp‐P (temperature predictability), Temp‐M (temperature contingency), Prep–Max (maximum monthly rainfall), Prep–Min (minimum monthly rainfall), Prep–Mean (average monthly rainfall), Prep‐C (rainfall constancy), Prep‐P (rainfall predictability), Prep‐M (rainfall contingency), flow (presence or absence), deep water (close <1 km or distant >1 km)

Fish were collected from four Lake Malawi habitats (Chisumulu island, Mbenji island, Mpatsonjoka dambo, and Makanjila) and one peripheral habitat (Linthipe river) in January 2011 (Figure 1, Table 1). Fish derived from a further three peripheral habitats (Enukweni, Ruvuma river, and Lake Chilwa) were taken from third‐generation stocks housed in the University of Hull (Figure 1). All fish were kept at a 12 hr: 12 hr light–dark regime and at water temperatures of between 25–28°C. The adults were fed once per day with King British tropical flake and juveniles with Interpet Liquifry No3 once a day. All tanks were equipped with UV and biological filters, aeration, synthetic aquarium foliage, and drainpipes of varying diameters that served as shelters.

**Table 1:** Source population sample sites, coordinates (decimal degrees), habitat, and experimental sample sizes

Population	Latitude °S	Longitude °E	Habitat	Experiment 1 number of clutches^a^	Experiment 2 number of broods^a^	Experiment 2 number of broods^b^
Chisumulu island	12.026	34.624	Lake Malawi (island)	18	16	10
Mbenji island	13.437	34.490	Lake Malawi (island)	15	16	12
Makanjila	13.693	34.848	Lake Malawi (lake margin)	17	16	12
Mpatsonjoka dambo	13.786	34.585	Lake Malawi (lake margin)	16	16	15
Enukweni	11.189	33.881	Peripheral habitat (swamp)	16	16	14
Linthipe river	14.177	34.126	Peripheral habitat (river)	16	16	16
Chilwa lake	15.371	35.591	Peripheral habitat (satellite lake)	18	16	14
Ruvuma headwaters	14.373	35.548	Peripheral habitat (river)	21	16	14

a Each clutch was from a different female, used for brood size, incubation time and fry length on release.

b Each clutch was from a different female, used to measure growth.

### 2.2 Environmental variables

Several variables were sourced for each sampling location, including water flow (flowing/not flowing), the proximity to water deeper than 20 m (close < 1 km, distant > 1 km) and altitude. Additionally, we derived interpolated monthly temperature and rainfall data from the CRU TS4.0 dataset at a resolution of 0.5° (Harris, Jones, Osborn, & Lister, 2014) for the period January 2001 to December 2015. We then used the “hydrostats” package in R (R Core Team, 2015; https://github.com/nickbond/hydrostats) to estimate environmental predictability (tightness of event to season), constancy (uniformity of event through all seasons), and contingency (repeatability of seasonal patterns), following Colwell (1974), using 10 bins of equal sizes in each calculation. To ordinate environmental similarity of sampling sites, we used a Principal Component Analysis based on a correlation matrix, in PAST 3.15 (Hammer, Harper, & Ryan, 2001).

### 2.3 Experimental populations

To ensure individual phenotypes were associated with parental genotypes, stock populations were bred to generate eight G1 (1st generation) populations that maximized available genetic diversity. From these G1 populations, experimental parental fish were drawn. Two separate experiments were performed, the first was aimed at testing differences in clutch size and egg mass among populations. The second was aimed at testing for differences among populations in brood size, brooding duration (incubation time), fry length when released, and juvenile growth rates.

### 2.4 Experiment 1

Response variables for the first experiment were clutch size (number of eggs), average individual egg size (g), and total egg investment (g). Between 15 and 21 G1 females from each of the eight populations were each mated with a single male from the same population (Table 1). Multiple females from the same population were housed in compartments with one male. Tanks were checked at least once daily for females that had spawned. Eggs were stripped from mouthbrooding females by gently pressing on their cheeks and opening and closing their mouth repeatedly. Once all eggs had been removed, each female was weighed using a Mettler Toledo PB602S balance, and the total length (TL) of the female was measured. The number of eggs in the clutch was counted and the eggs dried in an oven for 12 hr at 50°C. Eggs were then weighed on a Mettler Toledo AB54‐S balance.

### 2.5 Experiment 2

The second experiment used a hierarchical half‐sib design to quantify variation among populations and families (males). Response variables were incubation time (number of days from fertilization to release), brood size (number of fry released), and fry total length (at release, at day 35, at day 70). We generated second‐generation family clutches of offspring from four males, each mated with four different females, for each of the eight populations. In total we generated 128 families, and 1,595 individual offspring. This was achieved by housing females in a compartment with one dominant male. Tanks were checked at least once daily for any females that had spawned. Brooding females, easily identifiable by their pronounced gular, were removed from their tank and placed in 16.5 cm × 12.7 cm × 12.7 cm fry nets. We then checked daily to see if the female had released free‐swimming fry.

On release of fry, the mother was removed from the fry net, TL measured, and then weighed using a Mettler Toledo PB602S balance. We then calculated incubation time by counting the number of days from egg laying to fry release. The released fry were then placed in a water‐filled Petri dish, counted, and photographed with a size standard. They were then returned to their fry net. All broods were limited to 32 individuals, which were chosen at random. These fry were again photographed 35‐day postrelease, and 70‐day postrelease. Fry total length was measured using ImageJ 1.46 (Schneider, Rasband, & Eliceiri, 2012). Average brood growth rates were calculated as the difference in mean total length of fry in the brood between time points.

### 2.6 Genetic differentiation between sites

DNA was extracted from wild collected fish (Table 2 for sample sizes) using the Wizard^®^ DNA extraction kit (Promega Corporation, Madison, WI, USA). Samples were genotyped at seven microsatellite DNA loci: *Abur16*,* Abur46* (Sanetra, Henning, Fukamachi, & Meyer, 2009), *Ppun5*,* Ppun7*,* Ppun21*,* Ppun35* (Taylor et al., 2002), and *TmoM5* (Zardoya et al., 1996). Forward primers were labeled using 6‐FAM, NED, VIC, PET^®^ fluorescent dyes (Applied Biosystems, Inc., Foster City, CA, USA). All loci were amplified in the same multiplex reaction using the Qiagen multiplex PCR kit (Qiagen, Venlo, The Netherlands). The reaction contained 1 μl template DNA, 1 μl forward and reverse primer mix (2 pmol/L), 5 μl 2× Multiplex master mix (3 mmol/L MgCl_2_), and 3 μl RNase‐free water. PCR was performed in a BIO‐RAD MyCycler^™^ thermal cycler (Bio‐Rad Laboratories In., Hercules, CA, USA). Reactions consisted of an initial activation step of 15 min at 95°C, followed by 30 cycles of 30 s at 94°C, 90 s at 57°C and 60 s at 72°C and a final elongation step of 30 min at 60°C. PCR product was diluted 1 in 10 and GeneScan 500‐LIZ size standard added. Allele size was determined using an ABI 3500 genetic analyser (Applied Biosystems) and alleles called using GeneMapper 3.7 (Applied Biosystems).

**Table 2:** Genetic variability at seven microsatellite loci in seven populations of *Astatotilapia calliptera*. *n*, number of individual genotypes; *H*_E_, expected heterozygosity; *H*_O_, observed heterozygosity; *p*, significance of deviation from Hardy–Weinberg equilibrium

	*Ppun5*	*Abur16*	*Ppun7*	*Ppun35*	*Ppun21*	*Abur46*	*TmoM5*
Mbenji							
*n*	5	3	5	5	5	5	5
*H*_O_	1.000	0.667	0.600	0.400	0.200	0.400	0.600
*H*_E_	0.911	0.867	0.844	0.800	0.511	0.378	0.778
*p*	1.000	.466	.046	.029	.112	1.000	.190
Enukweni							
*n*	5	4	5	5	5	5	5
*H*_O_	1.000	1.000	0.800	0.800	1.000	0.800	0.200
*H*_E_	0.911	0.750	0.889	0.644	0.911	0.822	0.689
*p*	1.000	1.000	.612	1.000	1.000	.340	.048
Makanjila							
*n*	39	37	39	39	39	39	39
*H*_O_	0.974	0.919	0.974	0.949	0.923	0.821	0.821
*H*_E_	0.970	0.933	0.950	0.949	0.950	0.812	0.836
*p*	.744	.437	.593	.313	.824	.253	.495
Lake Chilwa							
*n*	25	25	26	26	26	26	26
*H*_O_	0.920	0.760	0.769	1.000	0.808	0.115	0.423
*H*_E_	0.940	0.859	0.942	0.928	0.912	0.113	0.474
*p*	.216	.248	<.001	.983	.123	1.000	.367
Ruvuma							
*n*	16	16	16	16	16	16	16
*H*_O_	0.313	0.688	0.875	1.000	0.875	0.125	0.063
*H*_E_	0.579	0.621	0.788	0.881	0.821	0.315	0.063
*p*	.001	.731	.824	.774	.284	.048	1.000
Linthipe							
*n*	13	9	13	13	13	13	13
*H*_O_	0.923	0.333	0.923	0.846	0.769	0.615	0.846
* H*_E_	0.926	0.673	0.938	0.898	0.926	0.542	0.806
*p*	.891	.003	.360	.843	.053	.768	.501
Chisumulu							
*n*	17	17	17	17	17	17	17
*H*_O_	0.941	0.824	0.941	1.000	1.000	0.353	0.824
*H*_E_	0.939	0.768	0.959	0.964	0.932	0.319	0.768
*p*	.898	.881	.716	1.000	.945	1.000	.166

### 2.7 Experimental data analysis

Response variables were analyzed using General Linear Models (GLMs) in R, with Tukey's HSD post hoc comparisons using an adjusted *p*‐value for multiple comparisons. Least‐square means of focal response variables, correcting for statistically significant covariables, were calculated using the package “lsmeans” (Lenth & Hervé, 2014). In Experiment 1, we focused on clutch size, average individual egg mass and total egg investment, using female postspawning TL as a covariate. We noted that female postspawning TL had a strong linear relationship with postspawning mass (*F*_1,136_ = 500.935; *r* ^2^ = .788; *p *<* *.001). In Experiment 2, we focussed on brood size, incubation time, average size of fry on release, using female postbrooding TL as covariate. Female postbrooding TL had a strong linear relationship with postbrooding mass (*F*_1,127_ = 1324.575; *r* ^2^ = .913; *p *<* *.001). We also considered growth between release and day 35, growth between release and day 70, in these cases using fry rearing densities for the relevant time periods as covariates. Comparisons of growth rates among populations were restricted to the 107 broods that contained between 5 and 19 individual offspring, inclusive, to reduce bias potentially introduced by variation in rearing density.

We used Principal Component Analysis in PAST 3.15 (Hammer et al., 2001) based on a correlation matrices to summarize associations between response variables measured for each brood in each experiment. From experiment 1, we used the standardized residuals of clutch size, average individual egg mass, and total egg investment from linear regressions against female TL. From experiment 2, we used we used incubation time, average fry TL of brood at release, standardized residuals of brood size regressed against female TL, and standardized residuals of average growth of each brood regressed against average fry density (log_10_ transformed) for the corresponding time period.

Response variables measured in experiment 2 were additionally analyzed using a linear mixed model approach within the “lme4” package in R (Bates, Maechler, Bolker, & Walker, 2015) to extract within‐population and between‐population variance components for the calculation of quantitative trait variation (*Q*_ST_). Population was considered a random effect of interest, with male identity set as a random factor nested within population. This enabled the direct estimation of between‐population variance (*V*_b_), and within‐population variation (*V*_w_) was calculated by adding the between‐male variation to the residual variation from the model. *Q*_ST_ values between every population pair were calculated using the following formula (Leinonen, McCairns, O'Hara, & Merilä, 2013; Sæther et al., 2007):

$$Q_{\mathrm{ST}}\;=\;\frac{V_{b}}{\left(2V_{w}\;+\;V_{b}\right)}$$

### 2.8 Genetic data analysis

Linkage disequilibrium among loci was quantified within populations using GENEPOP 4.2 (Raymond & Rousset, 1995), employing the log‐likelihood ratio statistic, 1,000 dememorizations, 100 batches, and 1,000 iterations per batch. Significant linkage disequilibrium was tested across locus pairs using Fisher's method, but no evidence was found. Observed and expected heterozygosity was calculated in Arlequin 3.5 (Excoffier, Laval, & Schneider, 2005). Deviations from Hardy–Weinberg equilibrium were calculated for each locus and each population using an Exact test with 1,000,000 steps in the Markov chain and 100,000 dememorization steps in Arlequin 3.5. The genetic relationships among populations were estimated using a pairwise *F*_ST_ distance matrix calculated in GENEPOP 4.2. Genetic distance among individuals was ordinated using a Principal Component Analysis in the “adegenet” package in R (Jombart & Ahmed, 2011). *Q*_ST_ values were compared with *F*_ST_ between source localities using a Spearman's rank permutation procedure in the “coin” package in R (Hothorn, Hornik, Van De Wiel, & Zeileis, 2008).

## 3 RESULTS

### 3.1 Environmental variables

Lake Malawi habitats were characterized by a close proximity to deep water, non‐flowing waters, relatively low attitude, warm temperatures, high precipitation, low predictability of temperatures, and high predictability of rainfall. By contrast, the peripheral habitats were characterized by absence of deep water, flowing waters, high altitudes, cold temperatures, high predictability, and contingency in temperature, but low predictability of rainfall (Figure 1c).

### 3.2 Experiment 1

**Figure 2:**
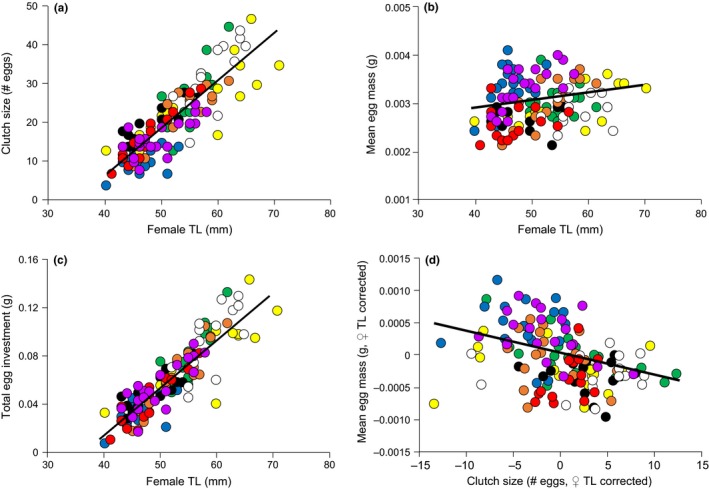
Experiment 1: Associations between female TL and (a) the number of eggs in a clutch, (b) the mass of individual eggs, and (c) the mass of the whole clutch (=total egg investment). After correcting for female length there is a trade‐off (d) between the number of eggs in a clutch and the mean mass of individual eggs in the clutch. Each point represents one clutch from one female. For population color codes see Figure 1

**Figure 3:**
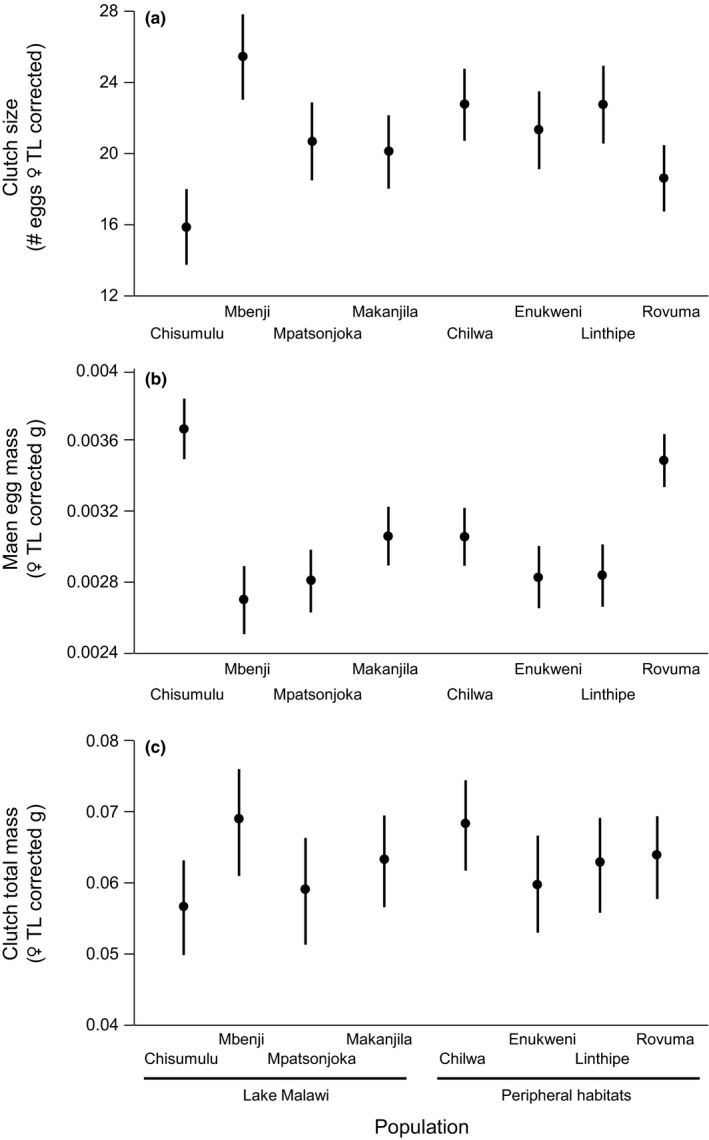
Experiment 1. Least‐square population means (95% confidence intervals) of measured life history traits. All variables shown are corrected for maternal female total length

Clutch size (# eggs) increased with female total length (TL; Figure 2a) and differed significantly among populations (Figure 3a; Table 3). Post hoc comparisons showed that Chisumulu clutches contained significantly fewer eggs than Chilwa, Enukweni, Linthipe, and Mbenji after correcting for female length. They also showed Mbenji clutches were larger than Makanjila and Ruvuma (Table 4). Average egg mass increased with female TL (Figure 2b) and differed significantly among populations (Figure 3a; Table 3). Post hoc comparisons showed that Chisumulu and Ruvuma eggs were larger than all other populations (Table 4). Total egg investment increased with female TL (Figure 2c) but did not significantly differ among populations (Figure 3c; Table 3).

**Table 3:** General linear models of differences among populations in Experiment 1

Response variable	Predictor variable	*df*	*F*	*p*
Clutch size (*n*)	Female TL (mm)	1,121	493.36	<.001
	Population	7,121	6.93	<.001
	Female TL × Population	7,121	2.88	.008
	Lake vs. peripheral	1,5	0.125	.738
Average egg mass (g)	Female TL (mm)	1,121	13.78	<.001
	Population	7,121	17.30	<.001
	Female TL × Population	7,121	2.44	.022
	Lake vs. peripheral	1,5	0.16	.905
Total egg investment (g)	Female TL	1,121	549.16	<.001
	Population	7,121	1.53	.161
	Female TL × Population	7,121	2.32	.030
	Lake vs. peripheral	1,5	0.442	.536

**Table 4:** Significance of differences between populations in *post hoc* comparisons of life history traits. Presented are Tukey's HSD *p*‐values adjusted for multiple comparisons. Bold indicates *p *<* *.05

Population 1	Population 2	Experiment 1		Experiment 2				
		Clutch size^a^	Mean egg mass^a^	Brood size^a^	Incubation time	Fry length release	Fry growth 0–35 days^b^	Fry growth 0–70 days^c^
Chilwa	Chisumulu	**<0.001**	**<0.001**	0.695	1.000	**<0.001**	**<0.001**	**0.029**
Chilwa	Enukweni	0.975	0.513	0.974	0.747	1.000	0.126	1.000
Chilwa	Linthipe	1.000	0.639	0.974	**0.001**	1.000	0.199	0.752
Chilwa	Makanjila	0.588	1.000	0.980	**0.024**	0.955	0.907	0.761
Chilwa	Mbenji	0.644	0.076	1.000	0.396	1.000	0.906	0.975
Chilwa	Mpatsonjoka	0.883	0.509	0.943	0.483	0.647	0.513	0.802
Chilwa	Ruvuma	0.077	**0.006**	0.999	0.993	1.000	0.999	0.947
Chisumulu	Enukweni	**0.020**	**<0.001**	0.190	0.662	**<0.001**	0.156	0.071
Chisumulu	Linthipe	**<0.001**	**<0.001**	0.127	**<0.001**	**<0.001**	0.065	0.500
Chisumulu	Makanjila	0.113	**<0.001**	0.147	**0.016**	**<0.001**	**0.007**	0.645
Chisumulu	Mbenji	**<0.001**	**<0.001**	0.540	0.317	**<0.001**	**0.006**	0.293
Chisumulu	Mpatsonjoka	0.026	**<0.001**	0.085	0.396	**0.001**	**0.018**	0.472
Chisumulu	Ruvuma	0.484	0.754	0.955	0.998	**<0.001**	**<0.001**	**0.001**
Enukweni	Linthipe	0.986	1.000	1.000	0.103	0.989	1.000	0.924
Enukweni	Makanjila	0.992	0.517	1.000	0.662	0.833	0.875	0.920
Enukweni	Mbenji	0.142	0.972	0.996	0.999	0.994	0.877	0.998
Enukweni	Mpatsonjoka	1.000	1.000	1.000	1.000	0.419	0.992	0.947
Enukweni	Ruvuma	0.605	**<0.001**	0.792	0.248	0.991	**0.029**	0.809
Linthipe	Makanjila	0.673	0.624	1.000	0.962	0.999	0.955	1.000
Linthipe	Mbenji	0.758	0.973	0.997	0.317	1.000	0.955	1.000
Linthipe	Mpatsonjoka	0.867	1.000	1.000	0.248	0.917	0.999	1.000
Linthipe	Ruvuma	0.075	**<0.001**	0.738	**<0.001**	1.000	**0.047**	0.118
Makanjila	Mbenji	**0.017**	0.084	0.998	0.930	0.998	1.000	0.999
Makanjila	Mpatsonjoka	1.000	0.490	1.000	0.883	0.998	0.999	1.000
Makanjila	Ruvuma	0.965	**0.007**	0.773	**0.002**	0.999	0.602	0.153
Mbenji	Mpatsonjoka	0.125	0.994	0.990	1.000	0.886	0.999	1.000
Mbenji	Ruvuma	**0.001**	**<0.001**	0.988	0.073	1.000	0.586	0.436
Mpatsonjoka	Ruvuma	0.829	**<0.001**	0.634	0.103	0.905	0.180	0.147

a Accounting for female TL.

b Accounting for mean fry density days 0–35.

c Accounting for mean fry density days 0–70.

There was a significant negative correlation between clutch size (# eggs) and average egg mass, after correcting for female TL (Pearson's *r *=* *−.393, *n *=* *137, *p *<* *.001; Figure 2d). There was no significant difference among populations in the association between mean egg mass (response variable) and number of eggs within those clutches (predictor variable), after correcting for female TL (GLM; *F*_7,120_ = 1.834, *p *=* *.087), indicating a common trait covariance across populations of the species. We found no significant differences between Lake Malawi and peripheral habitat populations in any variables (Table 3).

### 3.3 Experiment 2

**Figure 4:**
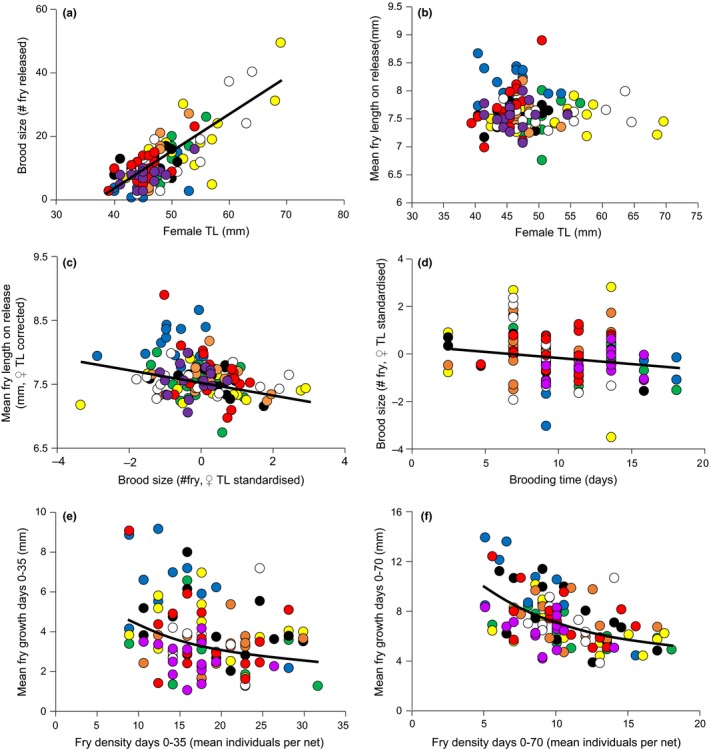
Experiment 2: Associations between: (a) brood size and maternal TL. (b) mean fry TL on release and maternal TL. (c) fry TL and brood size by standardized maternal TL. (d) incubation period and brood size by standardized maternal TL. (e) fry growth between days 0–35 in relation to fry density. (f) fry growth between days 0–70 in relation to fry density. For population color codes see Figure 1

**Figure 5:**
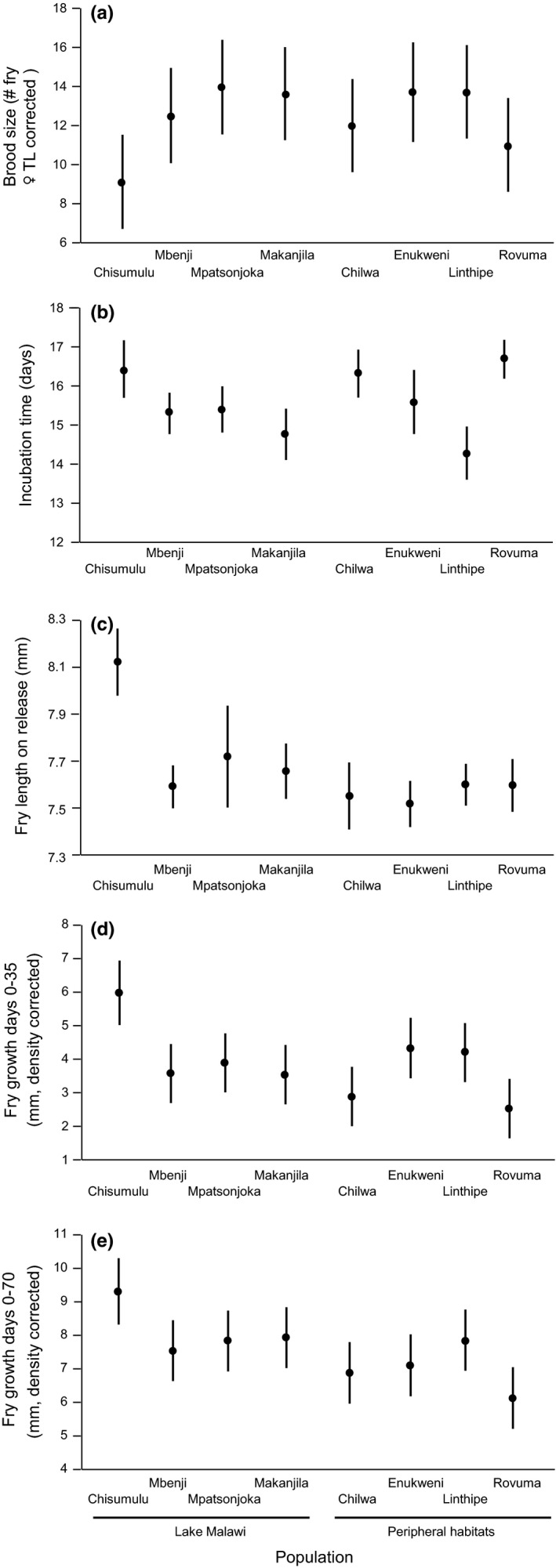
Experiment 2. Least‐square population means (95% confidence intervals) of measured life history traits. Brood size was adjusted for maternal female total length, while fry length at day 35 and 70 was corrected for rearing density

Brood size increased with female TL (Figure 4a) and differed among populations (Figure 5a; Table 5). However, no pairwise comparisons of populations showed significant differences in brood size (Figure 5a; Table 4). Incubation time was not dependent on female TL, and differed significantly among populations (Figure 5b; Table 5). Post hoc Tukey's HSD tests revealed that Chisumulu, Ruvuma, and Chilwa all had significantly longer brooding times than the Linthipe and Makanjila populations (Table 4). There was a significant negative correlation between incubation period and female TL‐corrected brood size (Pearson's *r *=* *−.195, *n *=* *128, *p *=* *.027; Figure 5d), but no significant correlation between the incubation period and average TL of fry released (Pearson's *r *=* *.084, *n *=* *128, *p *=* *.344). Mean TL of fry released was not dependent on female TL but did differ among populations (Table 5; Figure 5c). Pairwise comparisons showed Chisumulu fry were larger at the time of release than the fry of all the other sites examined (Table 4).

**Table 5:** General linear models of differences among populations in Experiment 2

Response variable	Predictor variable	*df*	*F*	*p*
Brood size (*n*)	Female TL (mm)	1,112	191.50	<.001
	Population	7,112	2.20	.039
	Female TL × Population	7,112	2.68	.013
	Lake vs. peripheral	1,5	0.005	.948
Incubation time (days)	Female TL (mm)	1,112	0.267	.606
	Population	7,112	6.518	<.001
	Female TL × Population	7,112	0.961	.463
	Lake vs. peripheral	1,5	0.160	.706
Fry TL on release (mm)	Female TL (mm)	1,112	1.062	.305
	Population	7,112	8.478	<.001
	Female TL × Population	7,112	1.501	.174
	Lake vs peripheral	1,5	2.133	.204
Growth days 0‐35 (mm)	Density (log_10_ individuals)	1,98	8.455	.005
	Population	7,98	7.045	<.001
	Lake vs. peripheral	1,5	0.047	.375
Growth days 0‐70 (mm)	Density (log_10_ individuals)	1,98	33.121	<.001
	Population	7,98	4.849	<.001
	Lake vs. peripheral	1,5	3.084	375

There was a significant negative correlation between average TL of fry released and female TL‐corrected brood size (Pearson's *r *=* *−.252, *n *=* *128, *p *=* *.004; Figure 4c). However, there was no significant difference among populations in the association between average TL of fry released (response variable) and number of fry within those clutches (predictor variable), after correcting for female TL (GLM; *F*_7,111_ = 0.307, *p *=* *.949), consistent with common trait covariance across *A. calliptera* populations.

Fry growth to day 35 was negatively related to the densities of individuals in compartments (Figure 4e), and differed among populations (Figure 5d; Table 5). Pairwise comparisons revealed that Chisumulu fry had grown more at day 35 than those from Chilwa, Makanjila, Mbenji, Mpatsonjoka, and Ruvuma (Table 4). Additionally, Ruvuma populations had grown less than populations from Enukweni and Linthipe (Table 4). Fry growth to day 70 was negatively related to the densities of individuals in compartments (Figure 4f), and differed among populations (Figure 5e). Pairwise comparisons revealed that Chisumulu fry had grown more to day 70 than those from Lake Chilwa and the Ruvuma river (Table 4).

A brood's average fry length on release and was positively related to the length achieved by day 35 (Pearson's *r *=* *.240, *n *=* *107, *p *=* *.013), and day 70 (Pearson's *r *=* *.30, *n *=* *107, *p *=* *.002).). However, average fry length on release was not significantly associated with either net growth between days 0 and 35 (Pearson's *r *=* *.070, *n *=* *107, *p *=* *.475) or days 0 and 70 (Pearson's *r *=* *.153, *n *=* *107, *p *=* *.116). We found no significant differences between Lake Malawi and peripheral habitat populations in any variables (Table 5).

### 3.4 Summary of trait covariance

**Figure 6:**
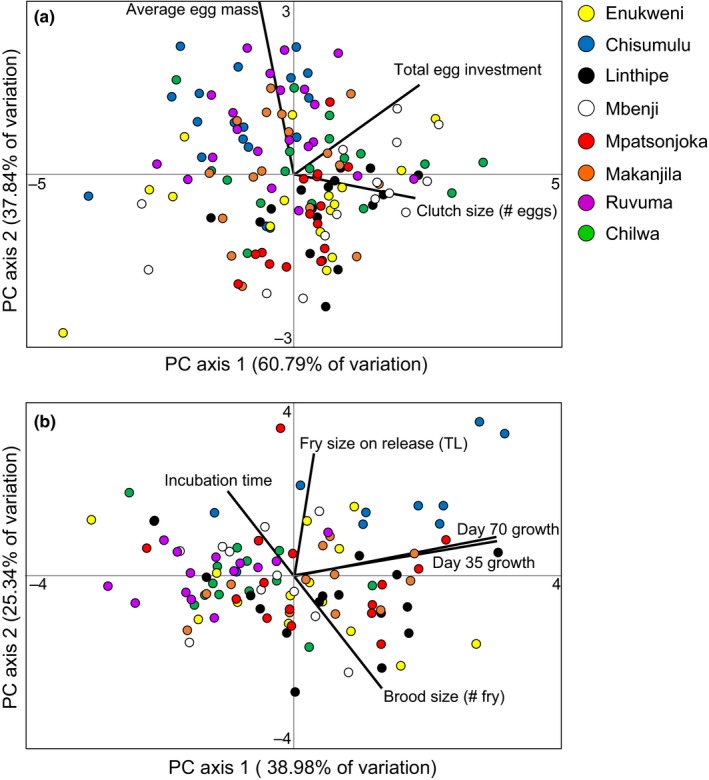
Principal component ordinations illustrating correlations between measured life history traits in (a) Experiment 1 and (b) Experiment 2

Principal component analysis response variable loadings (>0.4) from Experiment 1 showed a positive covariance between clutch size and total egg investment along PC axis 1, while average egg mass showed variation along PC axis 2 (Figure 6a).

Principal component analysis response variable loadings (>0.4) from Experiment 2 showed a positive covariance between growth to day 35 and growth to day 70 on PC axis 1. There was positive covariance between incubation time and brood's average fry length on release on PC axis 2 that both negatively covaried with brood size (Figure 6b).

### 3.5 Genetic differences among populations, and associations between *F*_ST_ and *Q*_ST_

Four of the 49 tests of deviation from Hardy–Weinberg Equilibrium were significant, but these were not consistent across populations or loci (Table 2). There was an overall significant genetic difference among populations (*F*_ST_ = 0.102; *p *<* *.001; Figure 1) with all population pairs differing significantly (*p *<* *.005) and *F*_ST_ values ranging from 0.039 to 0.266) (Appendix 1).

*Q*_ST_ was not significantly associated with *F*_ST_ for female size‐controlled brood size (Spearman's Rank; *Z *=* *−0.562; *p *=* *.581), incubation time (*Z *=* *0.629; *p *=* *.537), fry length on release (*Z *=* *−1.231; *p *=* *.227), density‐controlled growth rate to day 35 (*Z *=* *−1.086; *p *=* *.283), or density‐controlled growth rate to day 70 (*Z *=* *−1.147; *p *=* *.266). Pairwise comparison between *Q*_ST_ and *F*_ST_ indicated pairs of sites exhibiting both stabilizing selection (*F*_ST_ > *Q*_ST_) and directional selection (*Q*_ST_ > *F*_ST_) (Appendix 1).

## 4 DISCUSSION

Our experiments show that populations of *A. calliptera*, a cichlid in the Lake Malawi radiation, have diverged in key life history traits, including egg mass, clutch size, brooding time, and offspring growth rates. Although it is plausible that the differences may partly be due to transgenerational effects, our use of parent fish reared in the same laboratory conditions, suggests that the differences among populations cannot readily be attributed to nongenetic influences. Thus, we suggest that differences among populations most likely have a genetic basis, but it is also possible that unmeasured effects such as maternal age may have influenced the results. Additionally, it is possible that wild phenotypes are not reflected in the laboratory stocks, due to the potential for counter‐gradient variation operating on life history traits. Another consideration is that we employed *Q*_ST_‐*F*_ST_ comparisons, that can be informative for highlighting cases of selection (Leinonen et al., 2013), but can be subject to bias, such as in the mutation rate of molecular markers employed (e.g., Edelaar, Burraco, & Gomez‐Mestre, 2011).

Our source localities were classified broadly into Lake Malawi and peripheral habitats, on the basis of broad differences in environmental conditions. East African riverine environments are prone to strong seasonal and interannual fluctuations in water flow rate, and complete desiccation can take place (Dettinger & Diaz, 2000). By contrast, ancient lakes appear more predictable in depth and in temperature, nutrient regime, and oxygen content. The *r/K* model specifically addresses the role of environmental predictability in determining the evolution of life history strategy (Pianka, 1970; Winemiller & Rose, 1992). This model states that because unpredictable environmental change causes death irrespective of habitat quality, the most important determinants of lifetime reproductive success are survival to reproductive age and rapid reproduction, rather than competitive ability. Traits such as increased fecundity, large dispersal distance, fast growth rate, and early sexual maturity should, therefore, be favored in unpredictable habitats (Pianka, 1970). By contrast, in predictable environments, competitive ability and immunity/predator defences should be maximized because these environments are saturated by competitors, or parasites, predators, and pathogens (Parker & Begon, 1986). This means that traits such as maternal nutrient provision, gestation time, and parental care duration should be favored in predictable environments (Pianka, 1970). In our analyzes, however, we found no consistent differences in clutch size, egg size, or parental care duration between main lake and peripheral habitat populations, suggesting that spatial contrasts in life history traits are not necessarily related to habitat predictability, as may be expected under an *r*/*K* model.

### 4.1 Trade‐offs between offspring size and offspring number

Our results demonstrate a clear and consistent trade‐off between individual egg mass and clutch/brood size in *A. calliptera*. This is perhaps one of the best understood trait correlations, and it has been strongly linked to resource allocation (Messina & Fox, 2001; Smith & Fretwell, 1974). The trade‐off occurs because nutrients available to an organism are finite, and females must optimize allocation of these resources, resulting in a negative correlation between investment in individual offspring and the total number of offspring produced (Smith & Fretwell, 1974). In poor quality environments, females should favor a small clutch of highly provisioned offspring (Goulden, Henry, & Berrigan, 1987). By contrast, in high quality environments, females should produce larger clutches of less provisioned offspring because the greater resource availability within those habitats will allow offspring to compensate for initially small size through faster growth. It is possible that further work will identify the key limiting resources in the natural environment of the focal species, allowing tests to determine if environmental quality is a predictor of the provisioning strategy.

Alternately, or additionally, population differences offspring size and number may be a consequence of differences in age‐dependent among source locations. Investment in a small number of fry in habitats with high juvenile mortality may be risky, more so if a large proportion of mortality is unpredictable. Thus, the results demonstrating a diversity of investment strategies between egg number vs. egg size could be generated by differences in the temporal patterns of survivorship among the source localities. A closer understanding of the environmental variables associated with specific traits will require more detailed information on temporal patterns of survivorship across age classes within populations, and seasonal changes in resource availability.

In addition to life history trade‐offs, other factors could contribute to between population‐level differences in egg and clutch size, and may constrain adaptive divergence in response to local conditions. In mouthbrooding species such as *A. calliptera*, females store eggs in their mouths after fertilization, meaning that buccal volume imposes limits on clutch volume (Okuda, Tayasu, & Yanagisawa, 1998; Sefc, 2011). Buccal volume is in turn limited by gill size related to requirements for respiratory function (O'Connor, Reardon, & Chapman, 2012). Mouthbrooding capacity has also been linked to diet and head shape in haplochromine cichlids (Van Wassenbergh, Potes, & Adriaens, 2015; tkint, Verheyen, De Kegel, Helsen, & Adriaens, 2012). There is evidence that *A. calliptera* shows substantial differences in trophic resource use and head shape across its geographic range (P. Parsons, unpublished data). Taken together, it is possible that habitat characteristics such as oxygen concentration and food resource availability may also have driven the observed differences among populations.

### 4.2 Offspring size and growth

Our studies demonstrate a strong association between growth rates and rearing densities. This may be due to reduced food being available per individual, as food was not provided in overabundance. Alternatively it could be due to growth suppression induced by other aspects of the experimental conditions (Wedemeyer, 1997), including behavioral interactions among individuals. Such density dependent effects on growth may take place in natural conditions, but at present growth rates of wild fish are unclear. Insight into relative growth rates of fry in the natural environment could be gained from daily growth ring increments on otoliths (e.g., McLeod et al., 2015).

After controlling for density‐related effects on growth, we found that the length of fry on release was correlated with the size of fry after 35 and 70 days. However, we found no significant effect of the initial body size on net growth by days 35 and 70. This is suggestive of all populations exhibiting equivalent growth after the initial head start determined by maternal investment. Thus, our results do not support the model of Smith and Fretwell (1974), where the constraints of the trade‐off between offspring investment and number can be mitigated by smaller offspring having an increased growth rate (compensatory growth), enabling them to rapidly match the body size of more highly invested offspring. However, it is possible that initial maternal investment will affect multiple other offspring traits throughout their lifetime (Altmann & Alberts, 2004; Crean, Monro, & Marshall, 2011; Rius, Turon, Dias, & Marshall, 2010), and will not necessarily lessen in importance with age (Donelson, Munday, & McCormick, 2009; Heath, Fox, & Heath, 1999; Segers, Berishvili, & Taborsky, 2012).

### 4.3 Incubation time and fry length

Although we found significant differences in brooding duration among populations and that brooding duration tended to be longer in smaller broods, we found no significant relationship between incubation length and fry length. This was unexpected, given evidence that the Lake Tanganyika haplochromine cichlid *Ctenochromis horei* extends incubation period by approximately 4 days in the presence of predators and that this yields fry that are approximately 15% longer on release (Taborsky & Foerster, 2004). Incubation period may be related to aspects of fry morphology that were not assessed in this study. For example, we only measured fry length and it is possible that extra incubation yields higher body width and/or body mass (Gillooly, Charnov, West, Savage, & Brown, 2002), or other benefits such as increased immunity or brain development. Increased gestation associated with more extensive brain development has also been reported in mammals (Barton & Capellini, 2011; Sacher & Staffeldt, 1974). Also, since personality has been linked to several life history traits (Biro & Stamps, 2008; Niemela, Dingemanse, Alioravainen, Vainikka, & Kortet, 2013; Schuett et al., 2014; Wolf, van Doorn, Leimar, & Weissing, 2007), brood time may be related to particular personality traits that vary among *A. calliptera* populations. It is also not clear why incubation time should be negatively related to brood size. However, as smaller broods were persistently correlated with larger eggs and fry on release, these may require a greater duration of mouthbrooding.

### 4.4 Covariance among life history traits

Our results demonstrating trade‐offs and covariance between life history characteristics suggest that traits are not necessarily optimized by selection independently of each other, and that they can be intrinsically correlated in complex ways. In addition to correlations resulting from fundamental ecological trade‐offs driven by resource allocation, other factors such as genomic associations between traits that are driven by pleiotropy or linkage disequilibria may be important (Roff, 2007; Stearns, 1989). In the context of evolutionary divergence and adaptive radiation, such trait covariances could also prevent populations from reaching the adaptive peaks predicted by life history theory and local ecological conditions, and may therefore slow adaptive radiation more generally. It may be the case that persistent correlations between key traits across populations have constrained adaptive diversification (and specialization) between lacustrine or peripheral environments.

### 4.5 Rapid adaptive evolution in haplochromine cichlids

Exceptional rates of speciation and adaptive radiation of cichlids are often associated with strong selection on traits linked to sexual selection, habitat use or feeding ecology (Kocher, 2004; Malinsky et al., 2015; Wagner, Harmon, & Seehausen, 2012). The enhanced rates of adaptive radiation seen in lakes relative to surrounding rivers may be a consequence of both a complex adaptive landscape in lakes and that these landscapes persist for long enough (and populations are large enough) to permit adaptation in both ecological and sexually selected traits (Bridle & Jiggins, 2000; Seehausen, 2015). However, much less attention has been paid to selection on life history traits in cichlids, and how adaptive divergence in these traits is related to patterns of genetic population structure, structure, and stability of the immediate environment, as well as covariances among traits. Our results are consistent with the concept that genetic population structure is associated with divergence in life history traits, and in principle divergence of these populations may be promoted by selection acting against migrant genotypes linked to poorly adapted life history phenotypes.

The importance of life history evolution in rapid cichlid adaptive radiation is supported by comparative work on Lake Tanganyika and Lake Malawi cichlids, which demonstrates significant associations between individual egg mass and habitat use (Duponchelle et al., 2008), where pelagic species had larger eggs and lower fecundity than benthic and rock dwelling species. By contrast, our study provides no strong evidence for evolutionary divergence in life history traits among conspecific populations of cichlids linked to habitat, but it does provide evidence of persistent correlations among traits that in principle may limit the ability of populations of this focal species to reach adaptive peaks. Such constraints on adaptive diversification may partly explain why, uniquely among the Lake Malawi haplochromine species, *A. calliptera* retains a broad ecological niche, and is found in both riverine and lake habitats, despite their strongly contrasting ecologies. To more comprehensively understand the influence of environmental variability on life history trait evolution, we need more information on which traits covary, the underlying reasons for that covariance, and the extent to which such covariances promote or restrict rapid adaptive divergence in novel environments.

## CONFLICT OF INTEREST

None declared.

## AUTHOR CONTRIBUTIONS

MJG, LR, and JB conceived the research program. MJG, PJP, and JB conducted the fieldwork. PJP and MJG conducted the laboratory rearing trails, analyzed the data, and drafted the manuscript. All authors contributed the interpretation of data, revisions of the manuscript and approved the final version.

## DATA ACCESSIBILITY

Environmental, genetic and experimental data are on Dryad (https://doi.org/10.5061/dryad.6ps78).

## APPENDIX 1

### 1.1

**Table A1:** Comparison of pairwise genetic distances based on eight microsatellite markers (*F*_ST_) versus Quantitative trait differences (*Q*_ST_) measured in Experiment 2**.** *Q*_ST_ was calculated using the between‐population variance (*Vb*) and within‐population variance (*Vw*) as outlined in the methods

Population 1	Population 2	Trait	Between population	Within population	*F*_ST_ or *Q*_ST_	Population 1	Population 2	Trait	Between population	Within population	*F*_ST_ or *Q*_ST_
Chisumulu	Chilwa	*F*_ST_			0.098	Enukweni	Makanjila	*F*_ST_			0.057
		Brood size	3.340	14.104	0.106			Brood size	0.000	110.799	0.000
		Length fry at release	0.125	0.085	0.423			Length fry at release	0.012	0.045	0.115
		Growth days 0–35	3.982	2.676	0.427			Growth days 0–35	0.113	1.491	0.036
		Growth days 0–70	1.931	2.988	0.244			Growth days 0–70	0.000	2.837	0.000
		Incubation time	0.000	1.650	0.000			Incubation time	0.172	2.524	0.033
Chisumulu	Enukweni	*F*_ST_			0.159	Enukweni	Mbenji	*F*_ST_			0.159
		Brood size	4.534	54.190	0.040			Brood size	0.000	50.750	0.000
		Length fry at release	0.121	0.058	0.508			Length fry at release	0.000	0.042	0.000
		Growth days 0–35	0.300	2.958	0.048			Growth days 0–35	0.000	2.055	0.000
		Growth days 0–70	0.999	4.358	0.103			Growth days 0–70	0.000	2.957	0.000
		Incubation time	0.178	2.760	0.031			Incubation time	0.000	2.116	0.000
Chisumulu	Linthipe	*F*_ST_			0.110	Enukweni	Mpatsonjoka	*F*_ST_			NA
		Brood size	0.707	9.644	0.035			Brood size	0.000	42.930	0.000
		Length fry at release	0.103	0.051	0.504			Length fry at release	0.002	0.132	0.007
		Growth days 0–35	0.995	2.828	0.150			Growth days 0–35	0.000	2.641	0.000
		Growth days 0–70	0.000	4.385	0.000			Growth days 0–70	0.000	3.006	0.000
		Incubation time	2.594	2.058	0.387			Incubation time	0.000	2.146	0.000
Chisumulu	Makanjila	*F*_ST_			0.039	Enukweni	Ruvuma	*F*_ST_			0.266
		Brood size	28.750	90.970	0.136			Brood size	0.000	73.080	0.000
		Length fry at release	0.042	0.065	0.243			Length fry at release	0.000	0.047	0.000
		Growth days 0–35	2.779	2.414	0.365			Growth days 0–35	1.303	1.359	0.324
		Growth days 0–70	0.000	4.070	0.000			Growth days 0–70	0.000	2.270	0.000
		Incubation time	1.525	2.033	0.273			Incubation time	0.285	2.023	0.066
Chisumulu	Mbenji	*F*_ST_			0.065	Linthipe	Makanjila	*F*_ST_			0.055
		Brood size	4.320	11.640	0.157			Brood size	14.690	75.200	0.089
		Length fry at release	0.085	0.063	0.403			Length fry at release	0.005	0.039	0.061
		Growth days 0–35	1.620	3.456	0.190			Growth days 0–35	0.080	1.789	0.022
		Growth days 0–70	0.299	4.458	0.032			Growth days 0–70	0.000	3.480	0.000
		Incubation time	0.364	1.666	0.099			Incubation time	0.000	1.888	0.000
Chisumulu	Mpatsonjoka	*F*_ST_			NA	Linthipe	Mbenji	*F*_ST_			0.120
		Brood size	8.137	9.094	0.309			Brood size	0.000	10.850	0.000
		Length fry at release	0.024	0.163	0.070			Length fry at release	0.000	0.037	0.000
		Growth days 0–35	0.638	3.856	0.076			Growth days 0–35	0.000	2.307	0.000
		Growth days 0–70	0.000	3.930	0.000			Growth days 0–70	0.000	3.704	0.000
		Incubation time	0.497	1.745	0.125			Incubation time	0.699	1.647	0.175
Chisumulu	Ruvuma	*F*_ST_			0.165	Linthipe	Mpatsonjoka	*F*_ST_			NA
		Brood size	17.020	43.330	0.164			Brood size	1.742	8.708	0.091
		Length fry at release	0.097	0.067	0.420			Length fry at release	0.000	0.120	0.000
		Growth days 0–35	4.720	2.166	0.521			Growth days 0–35	0.000	2.791	0.000
		Growth days 0–70	3.817	3.239	0.371			Growth days 0–70	0.000	3.423	0.000
		Incubation time	17.020	43.330	0.164			Incubation time	0.584	1.711	0.146
Chilwa	Enukweni	*F*_ST_			0.139	Linthipe	Ruvuma	*F*_ST_			0.237
		Brood size	0.000	47.280	0.000			Brood size	8.425	35.560	0.106
		Length fry at release	0.000	0.062	0.000			Length fry at release	0.000	0.042	0.000
		Growth days 0–35	0.936	1.840	0.203			Growth days 0–35	1.252	1.579	0.284
		Growth days 0–70	0.000	1.961	0.000			Growth days 0–70	0.919	3.118	0.128
		Incubation time	0.102	2.257	0.022			Incubation time	2.927	1.457	0.501
Chilwa	Linthipe	*F*_ST_			0.149	Makanjila	Mbenji	*F*_ST_			0.062
		Brood size	0.000	10.851	0.000			Brood size	0.000	89.270	0.000
		Length fry at release	0.000	0.058	0.000			Length fry at release	0.000	0.049	0.000
		Growth days 0–35	0.752	2.073	0.154			Growth days 0–35	0.000	1.380	0.000
		Growth days 0–70	0.067	2.913	0.011			Growth days 0–70	0.000	3.005	0.000
		Incubation time	2.412	1.669	0.420			Incubation time	0.159	1.541	0.049
Chilwa	Makanjila	*F*_ST_			0.071	Makanjila	Mpatsonjoka	*F*_ST_			NA
		Brood size	6.048	83.880	0.035			Brood size	2.437	74.751	0.016
		Length fry at release	0.011	0.070	0.075			Length fry at release	0.000	0.132	0.000
		Growth days 0–35	0.090	1.494	0.029			Growth days 0–35	0.000	2.414	0.000
		Growth days 0–70	0.297	2.045	0.068			Growth days 0–70	0.000	2.872	0.000
		Incubation time	1.410	1.573	0.309			Incubation time	0.094	1.628	0.028
Chilwa	Mbenji	*F*_ST_			0.098	Makanjila	Ruvuma	*F*_ST_			0.156
		Brood size	0.000	13.695	0.000			Brood size	0.000	98.510	0.000
		Length fry at release	0.000	0.067	0.000			Length fry at release	0.003	0.053	0.032
		Growth days 0–35	0.086	2.102	0.020			Growth days 0–35	0.191	0.855	0.100
		Growth days 0–70	0.000	2.225	0.000			Growth days 0–70	0.920	2.275	0.168
		Incubation time	0.325	1.282	0.112			Incubation time	1.781	1.323	0.402
Chilwa	Mpatsonjoka	*F*_ST_			NA	Mbenji	Mpatsonjoka	*F*_ST_			NA
		Brood size	0.000	13.695	0.000			Brood size	0.000	9.094	0.000
		Length fry at release	0.000	0.067	0.000			Length fry at release	0.000	0.135	0.000
		Growth days 0–35	0.086	2.102	0.020			Growth days 0–35	0.000	2.915	0.000
		Growth days 0–70	0.000	2.225	0.000			Growth days 0–70	0.000	3.083	0.000
		Incubation time	0.325	1.282	0.112			Incubation time	0.000	1.277	0.000
Chilwa	Ruvuma	*F*_ST_			0.107	Mbenji	Ruvuma	*F*_ST_			0.237
		Brood size	5.734	39.000	0.068			Brood size	1.012	41.562	0.012
		Length fry at release	0.000	0.071	0.000			Length fry at release	0.000	0.049	0.000
		Growth days 0–35	0.000	1.284	0.000			Growth days 0–35	0.230	1.506	0.071
		Growth days 0–70	0.000	1.416	0.000			Growth days 0–70	0.430	2.466	0.080
		Incubation time	0.000	1.070	0.000			Incubation time	0.608	1.034	0.227
Enukweni	Linthipe	*F*_ST_			0.108	Mpatsonjoka	Ruvuma	*F*_ST_			NA
		Brood size	0.000	43.920	0.000			Brood size	0.604	35.630	0.008
		Length fry at release	0.000	0.036	0.000			Length fry at release	0.000	0.136	0.000
		Growth days 0–35	0.000	1.940	0.000			Growth days 0–35	0.457	2.334	0.089
		Growth days 0–70	0.000	3.675	0.000			Growth days 0–70	0.848	2.480	0.146
		Incubation time	0.805	2.496	0.139			Incubation time	0.709	1.183	0.230
